# Ecophysiological and ultrastructural characterisation of the circumpolar orange snow alga *Sanguina aurantia* compared to the cosmopolitan red snow alga *Sanguina nivaloides* (Chlorophyta)

**DOI:** 10.1007/s00300-020-02778-0

**Published:** 2020-12-11

**Authors:** Lenka Procházková, Daniel Remias, Andreas Holzinger, Tomáš Řezanka, Linda Nedbalová

**Affiliations:** 1 Department of Ecology, Faculty of Science, Charles University, Viničná 7, Prague, Czech Republic; 2 School of Engineering, University of Applied Sciences Upper Austria, Stelzhamerstr. 23, 4600 Wels, Austria; 3 Functional Plant Biology, Department of Botany, University of Innsbruck, Sternwartestraße 15, 6020 Innsbruck, Austria; 4 Institute of Microbiology, The Czech Academy of Sciences, Vídeňská 1083, 142 20 Prague, Czech Republic

**Keywords:** Arctic, Green algae, Astaxanthin, Polyunsaturated fatty acid, *Chlamydomonas nivalis*, Cryoflora

## Abstract

Red snow caused by spherical cysts can be found worldwide, while an orange snow phenomenon caused by spherical cells is restricted to (Sub-)Arctic climates. Both bloom types, occurring in the same localities at Svalbard, were compared ecophysiologically. Using a combination of molecular markers and light- and transmission electron microscopy, cells were identified as *Sanguina nivaloides* and *Sanguina aurantia* (Chlorophyceae). In search for reasons for a cosmopolitan vs. a more restricted distribution of these microbes, significant differences in fatty acid and pigment profiles of field samples were found. *S. aurantia* accumulated much lower levels of polyunsaturated fatty acids (21% vs. 48% of total fatty acids) and exhibited lower astaxanthin-to-chlorophyll-*a* ratio (2–8 vs. 12–18). These compounds play an important role in adaptation to extreme conditions at the snow surface and within snow drifts. Accordingly, the performance of photosystem II showed that one third to nearly half of the photosynthetic active irradiation was sufficient in *S. aurantia*, compared to *S. nivaloides*, to become light saturated. Furthermore, formation of plastoglobules observed in *S. nivaloides* but missing in *S. aurantia* may contribute to photoprotection. The rapid light curves of the two species show to a certain extent the shade-adapted photosynthesis under the light conditions at Svalbard (high *α*-value 0.16 vs. 0.11, low saturation point *I*_k_ 59 vs. 86). These results indicate significant physiological and ultrastructural differences of the two genetically closely related cryoflora species, but the reasons why *S. aurantia* has not been found at conditions outside (Sub-)Arctic climate types remain unknown.

**Supplementary Information:**

The online version of this article (10.1007/s00300-020-02778-0) contains supplementary material, which is available to authorised users.

## Introduction

Melting summer snowfields in polar regions are habitats for diverse microbial communities, including photoautotrophs causing snow discolorations (Hisakawa et al. 2015; Davey et al. 2019). Blooms of these microalgae significantly decrease snow and glacial surface albedo due to their pigmentation, thus, accelerating melting processes additionally to global warming (Lutz et al. 2016; Stibal et al. 2020). Red snow caused by spherical cysts, traditionally addressed as “*Chlamydomonas nivalis*” (regardless of their true taxonomic affiliation within the Chlorophyta), is probably the most common type of snow algal bloom in the Arctic and elsewhere. It has been intensely studied in the past (Hoham and Remias 2020). To a lesser extent, orange snow containing spherical cysts was reported from the Arctic as well (Leya et al. 2004; Stibal et al. 2007; Kvíderová 2012; Spijkerman et al. 2012). Recently, the algae of the two bloom types were described as distinct species: *Sanguina nivaloides* causing red snow and *Sanguina aurantia* causing orange snow. These species are not only distinguishable by the resulting macroscopic snow colour but also show differences in average cell sizes and in the sequence of the molecular ITS2 rDNA marker (Procházková et al. 2019a). No strains of *Sanguina* are available in culture, and thus, the life cycles of these algae, which should include migrating flagellates, are unknown. Practically, snow packs are populated by immotile, robust cysts during summer (Müller et al. 1998).

To understand the ecophysiological adaptation of these extremophiles to their harsh environment, characteristics such as life cycles, pigment composition, ice-binding proteins, photosynthetic light preferences and fatty acid profiles have been studied (Leya 2013). The focus of such studies has been on red snow forming species in high alpine (Remias et al. 2005) or polar sites (Soto et al. 2020), or on taxa causing orange snow in mid-latitude mountainous regions (Procházková et al. 2019b). In contrast, data on *S. aurantia*, which causes orange blooms at (Sub-)Arctic climate, are scarce.

While the red *S. nivaloides* has not only a striking cosmopolitan distribution in polar but also non-polar, mountainous regions, the orange *S. aurantia* has mainly been found in (Sub-)Arctic regions (Segawa et al. 2018; Procházková et al. 2019a) and recently in the alpine zone of mid-latitude mountain ranges of Northern America (Cascade Mts, Rocky Mts; Brown et al. 2016; Procházková et al. 2019a; Brown and Tucker 2020), where a local sub-arctic climate prevails (type Dfc sensu Köppen–Geiger climate system; Peel et al. 2007). The reasons for these biogeographic differences are unknown. Consequently, the aim of this study was, to our knowledge, the first attempt to compare physiological and ultrastructural traits of Arctic field communities to further elucidate the biogeographical patterns within this group, in particular the restriction of *S. aurantia* to Sub-Arctic and Arctic climate types. This comparison was performed by means of light- and transmission electron microscopy, photosynthesis measurements, analysis of pigments and fatty acids in *S. aurantia* and *S. nivaloides*. At Svalbard, blooms of both snow algae occurred at different spots in the same valleys (six cases) or in close proximity to each other (two cases), which made this comparative survey possible under practically identical abiotic conditions.

## Material and methods

### Sampling and snow characteristics

**Fig. 1:**
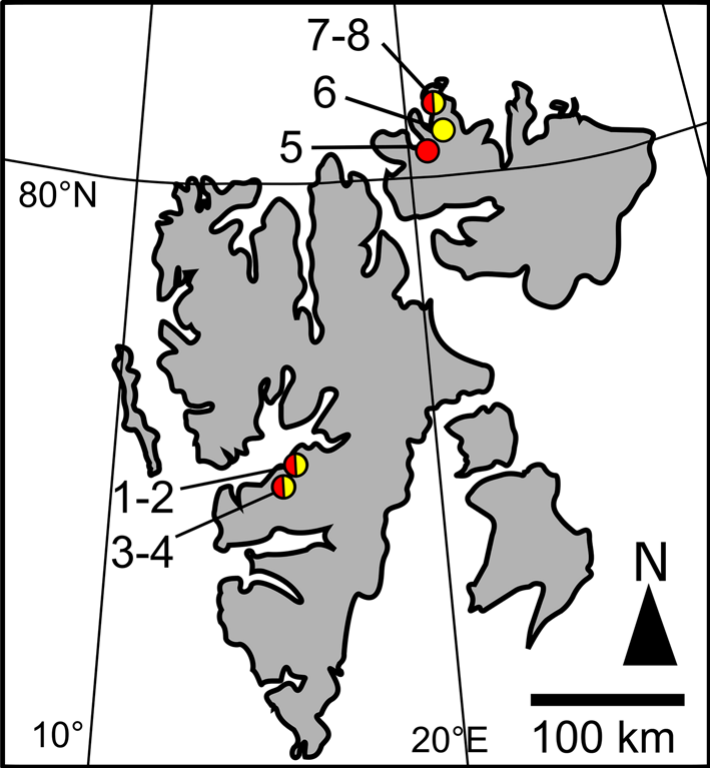
Snow sampling locations of *Sanguina nivaloides* (red circle), *Sanguina aurantia* (yellow circle) and where both species were harvested (red-yellow circles) at the archipelago of Svalbard. The indicated sample numbers refer to Table 1

In July 2018, orange and red booms of snow were investigated at Tverrdalen, Bjørndalen, Jaderinfjorden, Goosbukta and Laponia Halvøya, Svalbard, Norway (Fig. 1, Table 1). Virtually monospecific spots (one type of cells observed only) were intentionally selected based on light microscopy in the field to perform the polyphasic comparison between the two species, according to Procházková et al. (2019a). Four sites with a visible orange colouration and four sites with a red colouration were included in this study. Communities comprising a mixture of orange and red cells were excluded from the study. A surface snow layer of approximately 0.5 cm thickness was removed to reduce the content of dark snow detritus. Snow surface communities were harvested up to approximately 3 cm depth. Subsequently, orange and red samples were taken using a sterile steel scoop in 5-L buckets, 1-L thermos bottles or 50-mL centrifugation tubes, and transported the same day to the laboratory in Longyearbyen. The measurement of snow water equivalent (SWE; referred to as “snow water content” in Procházková et al. 2018a) was carried out as described previously (Procházková et al. 2018a). Prior to photosynthesis measurements, samples were melted gently at darkness overnight at 4–5 °C. Electrical conductivity (EC) and pH of the meltwater were obtained with WTW Instruments (Cond 340i and Inolab, Germany). To show the prevailing climate in the sampling region around Longyearbyen during the snow melting season, minimum, maximum and mean daily air temperatures (°C) were taken from a meteorological station in Adventdalen (78.202° 15.828°, 15 m a.s.l.).

**Table 1:** Sampling locations of *Sanguina* species in Svalbard (Norway) with sample codes, collection date, sampling site, altitude (metres a.s.l.) and geographic position (GPS)

Sample	Species	Date	Location	Altitude	GPS	Nr.
WP199	*S. aurantia*	3 July 18	Tverrdalen near Longyearbyen	293	N78 11.777 E15 31.325	(1)
WP204	*S. nivaloides*	5 July 18	Tverrdalen near Longyearbyen	365	N78 11.853 E15 31.012	(2)
WP206o	*S. aurantia*	7 July 18	Bjørndalen, west of Teltberget	347	N78 09.771 E15 18.315	(3)
WP206r	*S. nivaloides*	7 July 18	Bjørndalen, west of Teltberget	347	N78 09.771 E15 18.315	(4)
MN1	*S. nivaloides*	18 July 18	Jaderinfjorden, Lady Franklinfjorden	77	N80 07.825 E19 32.203	(5)
MN13	*S. aurantia*	19 July 18	Goosbukta, Brennevinsfjorden	6	N80 12.501 E19 48.101	(6)
MN21	*S. aurantia*	20 July 18	Laponia Halvøya, Brennevinsfjorden	127	N80 23.841 E19 38.957	(7)
MN22	*S. nivaloides*	20 July 18	Laponia Halvøya, Brennevinsfjorden	127	N80 23.841 E19 38.957	(8)

The numbers in parentheses indicate point of origin in the map of Fig. 1

### Light and transmission electron microscopy

**Fig. 2:**
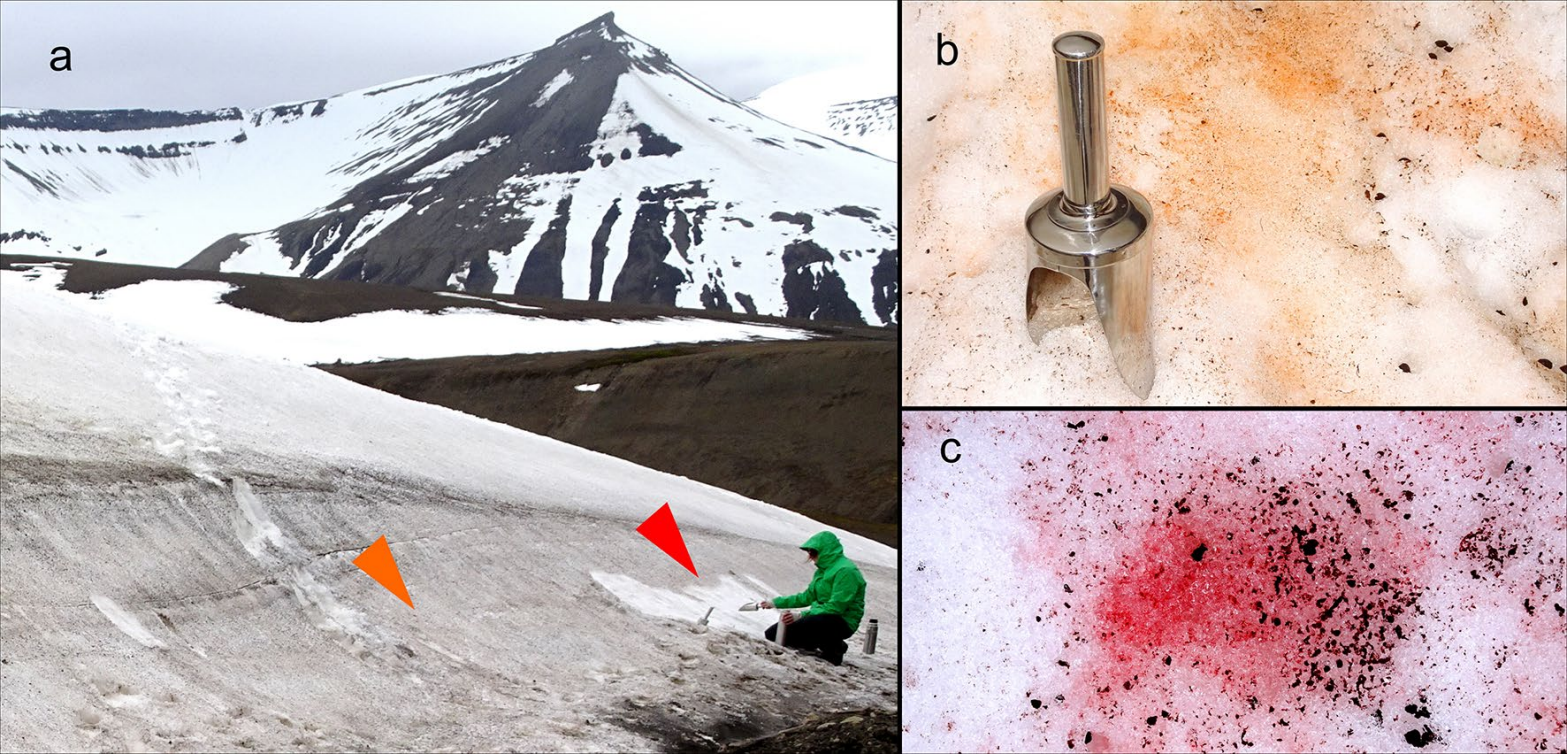
Overview of a sampling site in Bjørndalen, Spitsbergen, with blooms of *Sanguina aurantia* and *Sanguina nivaloides* (**a**) (samples WP206o, WP206r; bloom colours not visible from distance, marked by orange and red arrow, respectively), blackish colour was caused by snow detritus/cryoconite. Detail view of orange snow (**b**) caused by *S. aurantia* (sample WP199) and of red snow (**c**) dominated by *S. nivaloides* (sample WP204)

Morphological characterisation and determination of cell numbers per snow volume were performed by light microscopy (with an Olympus BX53 [equipped with an Olympus DP72 camera] or an Olympus BX43 [equipped with Nomarski Contrast and an Olympus DP27 camera or digital camera DXM 1200F (Nikon, Melville, NY, USA), using cellSens Entry Imaging Software]). Cell counting and transmission electron microscopy (TEM) of chemically fixed cells collected at WP199 and WP204 were carried out as previously described (Procházková et al. 2018a). The level of cyst maturation of less well-studied *Sanguina aurantia* (“young” vs. “mature”) was inferred from cellular changes observed during earlier studies for *Sanguina nivaloides* (late spring vs. late summer sampling): first, the astaxanthin-to-chlorophyll-*a* ratio raised during the ongoing season (Remias et al. 2005) and second, changes in the extent of intracellular lipid body organisation (compare fig. 2B–D vs. fig. 2A in Procházková et al. 2019a).

### DNA extraction, PCR and molecular analysis

DNA isolation was carried out with DNeasy Plant Mini Kit (Qiagen, Germany), as in Procházková et al. (2018a). The internal transcribed spacer region 2 (ITS2 rDNA) was amplified from DNA isolates by polymerase chain reaction (PCR) using existing primers SSU + LSU (Piercey-Normore and DePriest 2001) and ITS5 + ITS4 (White et al. 1990). Amplification reactions were described in Procházková et al. (2018a). PCR products were purified and sequenced using an Applied Biosystems-automated sequencer (ABI 3730xl) at Macrogen Europe (Amsterdam, Netherlands). The obtained ITS2 rDNA sequences were submitted to NCBI Nucleotide sequence database (accession numbers: WP199: MW202336; WP204: MW202335; WP206o: MW202276; WP206r: MW202275).

In this study, the term “haplotype” is used to refer to a unique ITS2 sequence within a species (Lutz et al. 2019). To check the species identity and assignment to the known haplotypes, ITS2 sequences obtained during this study were compared with available records at NCBI using BLAST search. A strict 100% sequence similarity threshold was applied (i.e. two sequences belong to the same haplotype if they are identical).

### Lipid extraction and fatty acid methyl esters analysis (FAMEs)

Cells were lyophilised for 48 h and stored frozen at − 80 °C prior to analysis. The extraction procedure was based on the method of Bligh and Dyer (1959), and elution was done from a Sep-Pak Vac Silica cartridge 35 cc (Waters; 10 g normal-phase silica) by chloroform (neutral lipids), acetone (glycolipids) and methanol (phospholipids) according to Saunders and Horrocks (1984). All classes of lipids were saponified overnight in 10% KOH in methanol at room temperature. The structures of FAMEs were assigned by comparison of Gas Chromatography/Mass Spectrometry retention times and fragmentation patterns with those of calibration standard FAMEs (Supelco, Prague), using methods of Temina et al. (2007) and Dembitsky et al. (1991). Procedures were described in detail at Procházková et al. (2018a).

### Pigment analysis

Cells were lyophilised for 48 h in darkness, extracted with methyl *tert*-butyl ether and the carotenoids and chlorophylls analysed by HPLC (Agilent 1200 ChemStation) using a C30 column (YMC Carotenoids) and a diode array detector set at 450 nm according to Procházková et al. (2019b). The peaks of *cis*-isomers of astaxanthin were assigned by the shift of the peak absorption maximum to lower wavelengths and the presence of an additional side absorption at approximately 375 nm, compared to the all-*trans* isomer used for calibration (Sigma Aldrich). Since the true dry mass of the snow algal field samples was not measurable due to impurities like cryoconite adhering to the cell walls, chlorophyll-*a* (chl-*a*) content was taken as a reference instead.

### Photosynthesis

In vivo chlorophyll fluorescence parameters of melted field samples were obtained with a pulse–amplitude modulated fluorometer (PAM 2000, HeinzWalz GmbH, Germany) in a 0.6-mL chamber and cooled with an ice bath to approximately 2 °C. To obtain the relative electron transport rates (rETR) and the light saturation point *I*_k_, cells were exposed to photon flux densities (PFD) of 5, 9, 34, 67, 104, 201, 366, 622 and 984 µmol photons m^−2^ s^−1^ for 30 s each. Four independent biological replicates were measured. For further details, see Procházková et al. (2018a).

### Statistical analyses

Values of average cells sizes, polyunsaturated fatty acids (PUFAs), content in total fatty acids (FAs), astaxanthin-to-chlorophyll-*a* ratio (astaxanthin-to-chl-*a* ratio) and relative content of geometrical stereoisomers of *cis*-astaxanthin (9Z/13Z astaxanthin) to all-*trans* astaxanthin of *S. aurantia* were compared with those of *S. nivaloides* using unpaired t test with Welch’s correction or the Mann–Whitney test in the programme Prism (GraphPad Software, CA, USA).

## Results

### Habitat conditions

During a field campaign in July 2018, several orange- and red-coloured, seasonal and semi-permanent snowfields were found at high Arctic in Svalbard. They occurred close to the coast and in inland valleys as well (Figs. 1, 2). The abiotic parameters of virtually monospecific blooms are summarised in Table 2. Meltwater had neutral or slightly acidic pH; its electrical conductivity was low (< 21 µS cm^−1^) and the water content in snowpacks (SWE) ranged between 50 and 60% at harvest. The regional climatic conditions during the polar summer included an air temperature drop below zero in mid-June (− 1 °C) and its peak at the beginning of August (13 °C) (Online Resource 1), the daily air mean temperature varied between 1.3 and 10.9 °C (Online Resource 2). From mid-June on, it rose above 5 °C.

**Table 2:** Abiotic habitat parameters and cyst sizes of *Sanguina* field samples from Svalbard

Species	Sample	EC	pH	SWE	Cells per mL meltwater	Cell diameter (µm) and size range (*n*)
*S. aurantia*	WP199	9.9	7.1	60.1 ± 4.7	167,500 ± 10,000	10.3 ± 1.5; 7.4–13 (30)
	WP206o	20.4	6.1	56.7 ± 3.5	122,300 ± 7400	13.8 ± 1.4; 10.5–16.3 (30)
	MN13	–	–	–	–	8.4 ± 1.3; 6–11.9 (30)
	MN21	–	–	–	–	10.3 ± 2.2; 6.4–14.3 (30)
*S. nivaloides*	WP204	12	7.0	59.5 ± 4.8	66,300 ± 5000	18.3 ± 5.2; 12.1–37.3 (30)
	WP206r	7.9	6.3	52 ± 3.3	102,450 ± 6700	14.5 ± 2.8; 9.9–23.2 (30)
	MN1	–	–	–	–	21.3 ± 3.7; 14.7–27.8 (20)
	MN22	–	–	–	–	20.2 ± 3.6.; 14–33 (30)

Electrical conductivities (EC; µS cm^−1^), pH of meltwater, the snow water equivalent (SWE; %), maximum population density ± standard deviation (SD), average cell sizes (µm) and cell size ranges (with *n* = number of cells measured) are shown

### Maximal population densities and cell morphologies

**Fig. 3:**
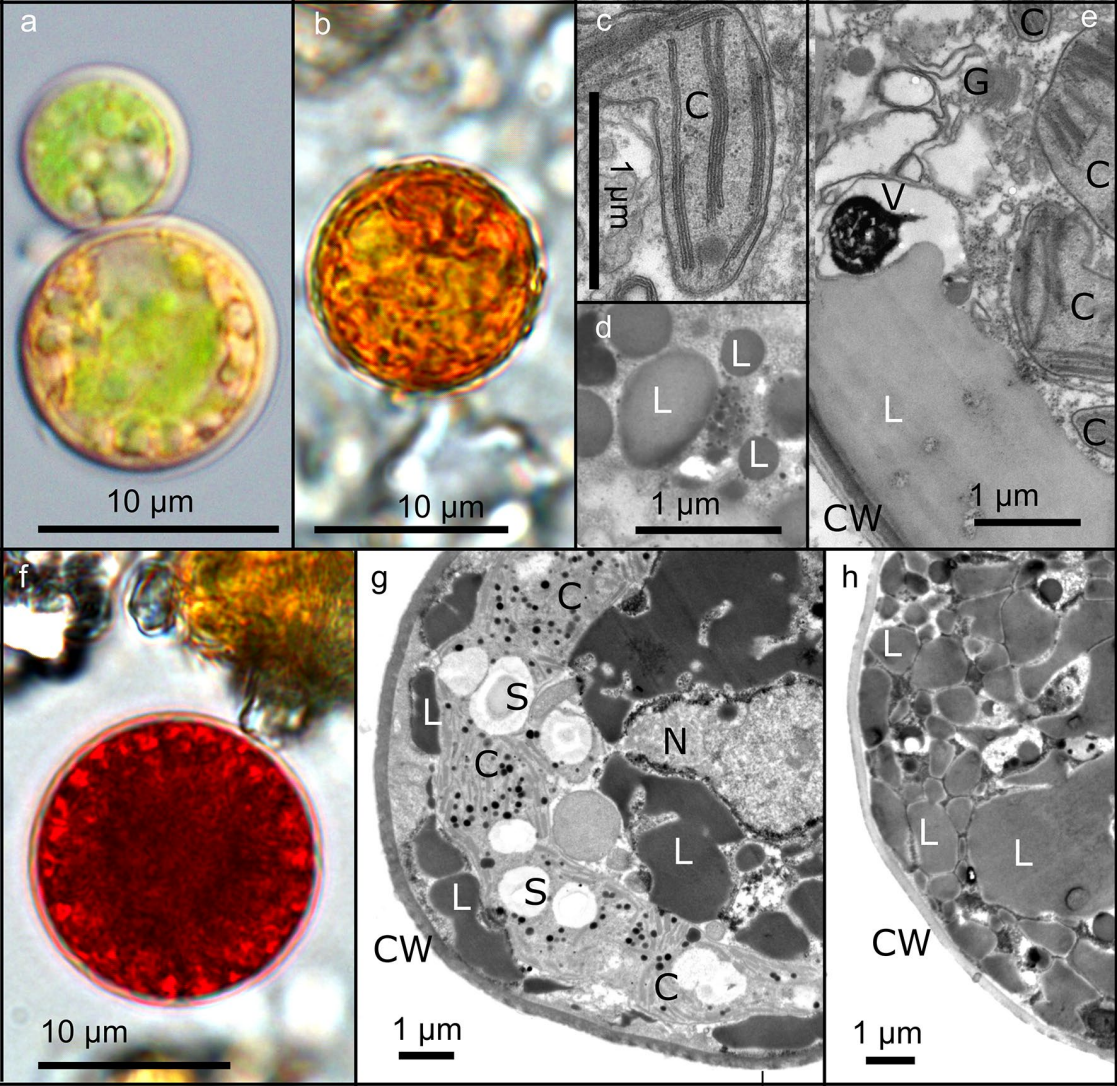
Light- and transmission electron micrographs of *Sanguina aurantia* (**a**–**e**; sample WP199) and *Sanguina nivaloides* (**f**–**h**; sample WP204) cysts collected in Svalbard. Young cysts with irregularly shaped chloroplast and small lipid bodies (**a**, **c**, **d**, **g**). Mature cysts with pronounced accumulation of lipid bodies (**b**, **e**, **f**, **h**). Note cell wall (CW), lipid body (L), starch grain (S) within the chloroplast (C), Golgi body (G), vacuole with electron dense crystalline content (V) and nucleus (N)

Four spots each of red and orange snow were compared. Cell sizes and cell number counts are summarised in Table 2. The maximal population densities of *S. aurantia* ranged from 1.22 to 1.67 × 10^5^ orange spherical cysts mL^−1^ and ranged from 0.66 to 1.02 × 10^5^ red spherical cysts mL^−1^ of *S. nivaloides*. The mean cell diameter of *S. aurantia* was 10.7 ± 2.6 µm (mean ± SD; n = 120), while it was 18.3 ± 4.7 µm (mean ± SD; *n* = 110) in *S. nivaloides* (Table 2). The cell sizes of the two species were significantly different (Mann Whitney test, *U* = 838.5, *p* < 0.0001 two tailed), and a marginal overlap of the cell size ranges was observed. No motile stages (flagellates) were observed for either species. In young cysts of *S. aurantia*, the cytoplasm contained an irregularly shaped and faint green chloroplast, and no other pigmented structures were present (Fig. 3a). Mature smooth-walled cysts of *S. aurantia* were dominated by orange pigmented lipid bodies (Fig. 3b). In this species, lipid bodies (Fig. 3d) characteristically fused during cyst maturation, resulting in a peripheral network of massive lipid accumulations (Fig. 3b, e). *Sanguina aurantia* possessed a low number of thylakoid membranes in the stroma of chloroplast lobes, forming loosely arranged layers, and no plastoglobules were visible (Fig. 3c, e). In young cysts of *S. nivaloides*, the parietal chloroplast containing numerous plastoglobules was visible (Fig. 3g). In mature smooth-walled cysts of *S. nivaloides*, lipid bodies fused as well (Fig. 3f, h). Prominent vacuoles containing an osmiophilic crystalline structure were found in both species (shown for *S. aurantia*, Fig. 3e).

### Molecular ITS2 rDNA haplotype identification

The taxonomical assignment of the samples based on cell morphology of *S. aurantia* and *S. nivaloides* was further confirmed by using the ITS2 rDNA molecular marker. The orange and red populations (accession numbers in “Material and methods” section) were genetically identical with the most common ITS2 rDNA haplotypes “HA1” and “H1”, respectively, as recently established for *Sanguina* (Procházková et al. 2019a).

### Fatty acid composition

**Fig. 4:**
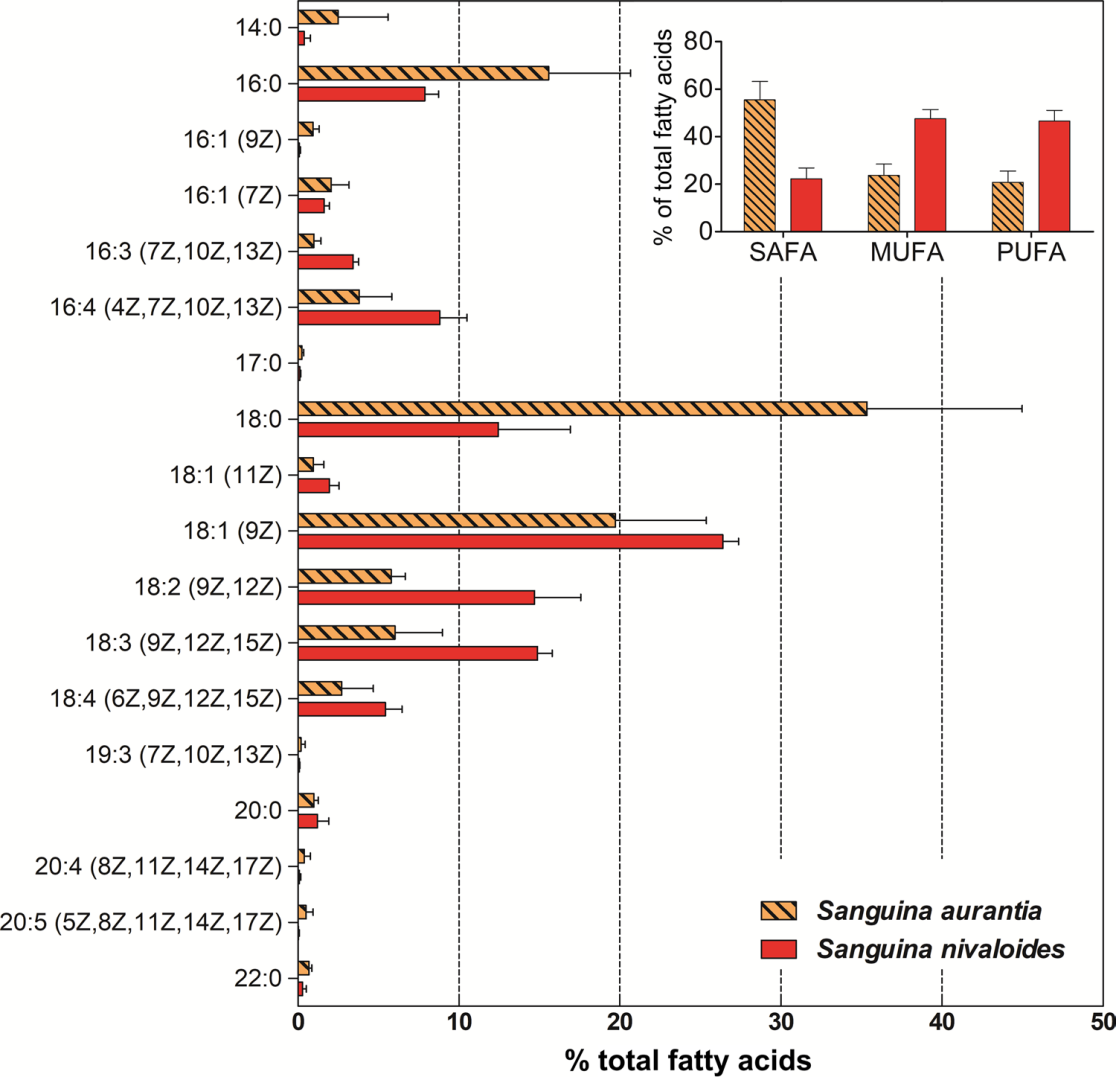
Average cellular fatty acid composition of *S. aurantia* (*n* = 4) and *S. nivaloides* (*n* = 3) field samples in (%) of total fatty acids (± standard deviation). The relative proportion of saturated (SAFA), monounsaturated (MUFA) and polyunsaturated (PUFA) fatty acids (± standard deviation) is given in the inset. The figure shows only fatty acids with abundances greater than 0.2%. A detailed fatty acid profile of all field samples including FAs accounting at least of 0.1% in total fatty acids is in Online Resource 3

The relative content of FAs (as % of total fatty acids) in samples of *S. aurantia* (*n* = 4) and *S. nivaloides* cysts (*n* = 3) is given in Fig. 4 (in more detail shown in Online Resource 3). FAs sizes ranged from 14:0 to 23:0. Both species accumulated PUFAs in significantly different quantities (unpaired t test with Welch’s correction, *Welch corrected t*_4_ = 7.006, *p* = 0.00222), about half in *S. aurantia* (20.8 ± 4.6%) than in *S. nivaloides* (47.6 ± 4.7% of total lipids; Table 3). The main monounsaturated fatty acid (MUFA) was oleic acid (18:1 (9Z); 19.7 ± 6.5% vs. 26.4 ± 1%). The major PUFAs for both species were α-linolenic acid (18:3 (9Z, 12Z, 15Z); 6 ± 3.4% vs. 14.9 ± 1.1%), followed by linoleic acid (18:2 (9Z, 12Z); 5.8 ± 1% vs.14.7 ± 3.5%), hexadecatetraenoic acid (16:4 (4Z,7Z,10Z,13Z); 3.8 ± 2.3% vs. 8.8 ± 2.1%) and stearidonic acid (18:4 (6Z, 9Z, 12Z, 15Z); 2.7 ± 2.2% vs. 5.4 ± 1.2%). The most prominent saturated fatty acids (SAFAs) were palmitic (16:0) and stearic acids (18:0).

**Table 3:** Relative amounts of lipid fractions in *S. aurantia* (WP199) and *S. nivaloides* (WP204) field cysts

	*Sanguina aurantia*	*Sanguina nivaloides*
Neutral lipids	55.0	72.7
Glycolipids	28.3	15.0
Phospholipids	16.7	12.3

Values are in (%) of total lipids

**Fig. 5:**
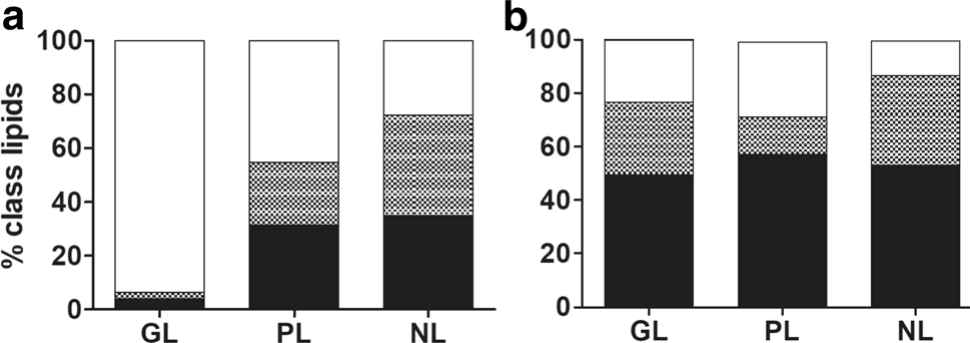
The relative proportions of saturated (white), monounsaturated (black-white checked) and polyunsaturated fatty acids (black) in *Sanguina aurantia* (**a**; sample WP199) and *Sanguina nivaloides* cysts (**b**; sample WP204) in the three main lipid classes (*GL* glycolipids, *PL* phospholipids, *NL* neutral lipids)

Neutral lipids predominated over glycolipids and phospholipids in total lipids in *S. aurantia* and *S. nivaloides* field samples (Table 3). There were no significant qualitative differences in FAs between the three major lipid classes of *S. nivaloides*: PUFAs dominated all glycolipids, phospholipids and neutral lipids. In contrast, saturated FAs strikingly prevailed in the glycolipids and were abundant in phospholipids in *S. aurantia* (Fig. 5, Online Resource 3).

### Pigment composition

The chlorophyll and carotenoid contents of the two species were compared to understand how the different colours of orange and red snow developed. By far, the most abundant pigment in all samples was the secondary keto-carotenoid astaxanthin, which composed 72 ± 9.9% (*n* = 4) and 91.7 ± 0.9% (*n* = 4) of all pigments in *S. aurantia* and *S. nivaloides*, respectively. Depending on the stage within cyst maturation, the field populations had an overall astaxanthin-to-chl-*a* ratio range from 2:1–8:1 for *S. aurantia*, and 12:1–18:1 for *S. nivaloides*. The astaxanthin-to-chl-*a* ratio was significantly different between the two species (unpaired t test with Welch’s correction, *Welch corrected t*_*5*_ = 5.830, *p* = 0.0021 two tailed). The HPLC chromatograms showed several peaks with identical visible absorption spectra, indicating the presence of astaxanthin derivative-like esters, and the retention times of these peaks were practically identical for both species (data not shown). About 5.4 ± 0.9% (*S. aurantia*; *n* = 4) and 8.1 ± 1.2% (*S. nivaloides*; *n* = 4), respectively, were recognised as native *cis*-isomers (likely 13*Z*) with a shorter wavelength of the absorption maximum and an additional side absorption in the UV range. The relative content of *cis*-isomers to all-*trans* astaxanthin was significantly different between the two species (unpaired t test with Welch’s correction, *Welch corrected t*_*5*_ = 3.162, *p* = 0.0250 two tailed). Further principal compounds of *S. aurantia* (*n* = 4) and *S. nivaloides* (*n* = 4) were chlorophyll-*a* and *b* (23.8 ± 8% and 7.3 ± 0.9% of all pigments, respectively) and lutein (5 ± 2.6% and 1 ± 0.4% of all pigments, respectively). In summary, orange snow contained less red (astaxanthin) but more yellow (lutein) and green compounds (chlorophylls).

### Photosynthesis

**Fig. 6:**
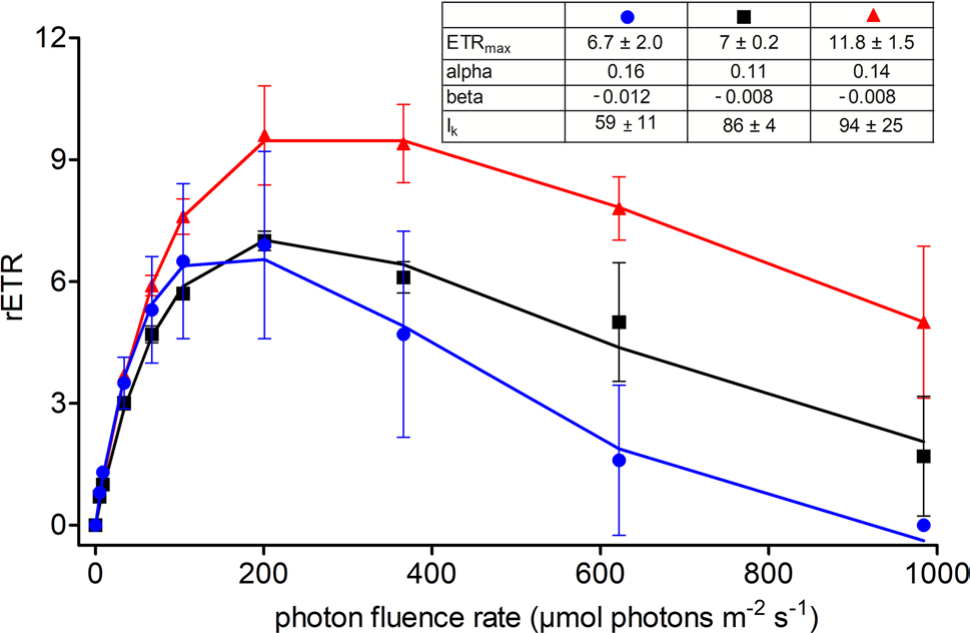
Comparison of three rapid light curves between field cysts of the two closely related *Sanguina* species. The effect of increasing photon fluence rates (*x*-axis) on the relative electron transport rate (rETR; *y*-axis) of chloroplasts are shown for *S. aurantia* (blue circles, sample WP199) and *S. nivaloides* (black boxes, sample WP206r; red triangles, sample WP204). Values are means of four independent biological replicates (*n* = 4, ± SD). The data points were fitted to the photoinhibition model of Walsby (1997)

The photosynthetic activities of *S. aurantia* and *S. nivaloides* were compared, and rapid light curves were generated (Fig. 6). The values of fluorescence parameters given below are the mean of four independent biological replicates. *S. aurantia* from south-eastern exposed Tverrdalen showed an alpha value of 0.16, a beta value of − 0.012, a relative ETR_max_ of 6.7 ± 2 and *I*_k_ of 59 ± 11 µmol photons m^−1^ s^−1^ (*n* = 4). By contrast, a *S. nivaloides* population occurring in the same valley showed an alpha of 0.14, a beta of − 0.008, a relative ETR_max_ of 11.8 ± 1.5 and *I*_k_ of 94 ± 25 µmol photons m^−2^ s^−1^ (*n* = 4). A population of *S. nivaloides* from a north-western exposure in Bjørndalen exhibited one quarter lower values to the other *S. nivaloides*: an alpha of 0.11, a relative ETR_max_ of 7 ± 0.2, while only slightly lower *I*_k_ of 86 ± 4 µmol photons m^−2^ s^−1^ and an identical beta of − 0.008 (*n* = 4). Thus, the light-dependent photosynthetic rates of *S. nivaloides* from these two sites suffered photoinhibition from medium light intensities (of the measured range) onwards, which was 400 or 200 µmol photons m^−2^ s^−1^. *S. aurantia* showed signs of photoinhibition from 200 µmol photons m^−2^ s^−1^ on.

## Discussion

### Ecology and spatial distribution

*Sanguina aurantia* and *S. nivaloides* thrived in melting summer snow at the same Arctic sites next to each another or alone not only in the same, but also in different valleys. At the scale of a dozen centimetres to a metre, a continuum of solely red or orange patches to occasional mixed spots consisting of the two species was observed, with mixed patches likely as a result of peripheral merging of populations in the course of snow melting (see Supplementary Fig. S2 in Procházková et al. 2019a). Alternatively, the orange cells were part of the macroscopically dark red population or vice versa, likely because “seed banks” of the two species (i.e. remaining cysts from the year before) were situated close to each other. In the metagenomic study of Brown et al. (2016), sun-exposed patches were composed of multiple species, and each species within a patch was highly clonal. It was inferred to be an effect of stochastic (re-)establishment of populations in situ from the previous growing seasons and strong founder or priority effect (i.e. the impact that an organism has on community development due to a sooner arrival at a site). Although is it not known at which point the blooms become macroscopically visible at Svalbard, the last air temperature measured below the freezing point occurred at least 2 weeks prior sampling. The cells reached maximum population densities comparable to those reported earlier from the same archipelago (Müller et al. 1998), namely 10^4^ cells mL^−1^ for either the orange (*S. aurantia*) or 10^5^ cells mL^−1^ for the red (*S. nivaloides*) spherical cysts. At lower cell densities (data not shown), snowfields dominated by the latter may appear pinkish (see the edges of the red spot in Fig. 2c). Other snow algae like *Chloromonas* (*C*.) spp. were very sparse and did not contribute significantly to these numbers (data not shown). One population in the present study (WP199, Table 1) was located in exactly the same position (identical GPS coordinates) as a sample collected 8 years earlier (Sva 10-8, Procházková et al. 2019a). As already observed in 2010, orange and red spots were close to each other in 2018. This is an indication that snow algae show a long-term site fidelity in such seasonal snowpacks, and this has been observed for other snow algae as well, e.g. *Chlainomonas* sp. (Remias et al. 2005; Procházková et al. 2018b). Other concepts including an annual airborne cell circulation have been proposed for persistent snow fields in steep snow slopes of Svalbard (Müller et al. 2001), which would explain a repeated observation of snow blooms year after year at these topographically prominent sites.

### Morphology of the cysts

The range of cell sizes described in the present study was consistent with those given in the initial descriptions of the two species in Procházková et al. (2019a). In this study, young cysts of both species significantly differed in their cytosolic lipid body organisation, fusing sooner during cell maturation into larger, irregularly shaped lipid bodies in *S. aurantia* (Fig. 3e) when compared to *S. nivaloides*. Lower numbers of thylakoid membranes per stroma volume and less thylakoids stacked to each other are a strategy of the highly dynamic photosystem II to respond to high light conditions (e.g. Anderson et al. 1988, 2008). This could explain the faint green colour of the chloroplast in *S. aurantia* at the light microscope. In contrast, an alpine population of mature red spherical cysts had 3 to 7 thylakoid membranes arranged in a grana-like arrangement in the central chloroplast, which was fully surrounded by abundant astaxanthin lipid bodies (Remias et al. 2005). Moreover, a striking difference was observed by TEM, while *S. aurantia* did not contain plastoglobules in the chloroplasts, these structures were very dominant in *S. nivaloides* chloroplasts, suggesting a different physiological status. Plastoglobules are lipoprotein particles inside the chloroplast accumulating upon high light stress (Austin et al. 2006; Rottet et al. 2015) and were prominently found upon experimental UV-A and UV-B treatments in the freshwater red alga *Batrachospermum turfosum* (Aigner et al. 2017) and the streptophyte green alga *Zygnema* sp. (Holzinger et al. 2018).

A convergent cellular adaptation for tolerating excessive irradiances was reported for another, ecologically and taxonomically different extremophilic algal group, the glacial algae: these microorganisms live in a distinct habitat, wet ice surfaces, but not in melting snow, and they are streptophytic algae and thus closer related to land plants than green algae sensu stricto where *Sanguina* belongs to. The chloroplast of glacial algae is positioned under the shading phenolic pigment and remains practically low-light-adapted despite exposure to full irradiation (Williamson et al. 2020). In these two cases, the strategy of protection by secondary pigments was realised by biochemically distinct compounds: the dark phenols of glacier algae are hydrophilic and stored in vacuoles, while the non-polar lipophilic carotenoids of red snow algae are accumulated in lipid bodies. These pigments have significant capabilities in absorbing UV and VIS wavelengths in both cases, despite with some spectral differences (Remias 2012).

### Fatty acid composition

In *S. nivaloides*, the content of PUFAs was similarly high in all lipid classes, and they were less abundant in *S. aurantia*. These compounds are important for tuning cellular responses and resilience under a range of harmful abiotic conditions and for the adjustment of the photosynthetic apparatus (Kugler et al. 2019). In accordance to this study, such high contents of PUFAs found in *Sanguina nivaloides*, in particular C18:2, C18:3, C18:4, C16:4 and C16:3, were reported from red snow dominated by the same species (the operation taxonomic unit [OTU] was assigned to “uncultured Chlamydomonadaceae” GU117577—now *S. nivaloides* in NCBI) in the study of Lutz et al. (2016). Moreover, a similarly high content of PUFAs was observed in red cysts of *Chlainomonas* and *Chloromonas* (Procházková et al. 2018b, 2019b), and for vegetative (green) flagellates of *Chloromonas* (Řezanka et al. 2008). In the present study, *Sanguina aurantia* accumulated only half the amount of PUFAs in total fatty acids as *S. nivaloides*. However, Spijkerman et al. (2012) reported a high Arctic population of spherical orange cysts harvested in mid-August (i.e. 1 month later than conducted here) to have a very high accumulation of PUFAs comparable with *S. nivaloides* from the same location (compare sample 9/10-1a and 9/10-1b in Spijkerman et al. (2012)), but their data were based on a single measurement. One may hypothesise that either the complete maturation of the cysts of *S. aurantia* takes longer (i.e. to accumulate very high levels of PUFAs), or *S. aurantia* is not a *true* snow alga sensu Remias (2012) (i.e. it does not reproduce *only* in meltwater between snow or ice crystals). The former hypothesis may be tested by a later field sampling. The second requires terrestrial sampling outside of snow fields. Both scenarios may partly explain the restricted distribution of *S. aurantia* in the (Sub-)Arctic climate which are present also in alpine zone of some mid-latitude mountain ranges (Peel et al. 2007). Interestingly, *Sanguina* lineages exist, which are different to *S. aurantia*, which seem to have a distribution restricted to one of the Earth’s poles: “*Chlamydomonas*”—snow group A sensu Segawa et al. (2018) (a member of a well-supported ITS2 rDNA clade comprising also *Sanguina nivaloides* GU117577.1) is common in Antarctica but almost absent in the Arctic in the data of Segawa et al. (2018). Investigation of special structuring of genetic diversity enables to document patterns in evolutionary divergence of eukaryotic organisms: some new microalgal lineages were shown to be biogeographically much more differentiated than old ones (Škaloud et al. 2019) while they probably do not differ in terms of possible dissemination.

The dominance of oleic acid 18:1 (9Z) in nutrient depleted laboratory cultures and in field cysts (Spijkerman et al. 2012) during late summer apparently reflects its importance as a precursor in the synthesis of PUFAs (e.g. linoleic and/or α-linolenic acids) in all eukaryotic organisms (Gurr 1971; Leya 2013). It means that high production of PUFAs is determined by the presence of oleic acid. Temperature modulates the fatty acid profile in Antarctic ice microalgae (An et al. 2013), and increased unsaturation of the fatty acids in membrane lipids plays a major role in avoiding membrane rigidification at low temperatures (Morgan-Kiss et al. 2006). Moreover, the biosynthetic pathway leading to oleic acid was highly upregulated in a green alga in a response to cold (Hwangbo et al. 2014). Oleic acid is also accumulated in the pre-akinete stages of *Zygnema* sp. derived from nitrogen depleted cultures or mature field samples (Pichrtová et al. 2016; Arc et al. 2020); therefore, this can be considered as a general strategy observed in phylogenetically distinct streptophytic organisms.

The key factors influencing the qualitative fatty acid composition of lipids are temperature, nutrients, PAR, taxonomic affiliation and the physiological state of an alga (Kumari et al. 2013). Since both bloom types were harvested from almost identical locations, the differences found should reflect species-specific responses (Teoh et al. 2004) or the cell maturity level at the time of harvest rather than site-specific microhabitat conditions. Interestingly, there is an increasing evidence that some algae isolated from polar habitats (snow, soil, sea water) have broad temperature optima for growth (covering mesophilic conditions), and SAFAs may dominate their FAs regardless of the cultivation temperature (Teoh et al. 2004). In general, molecular mechanisms behind adaptation of polar microalgae can be explored by a variety of “omic” techniques (Lyon and Mock 2014).

### Pigments

The ratio of astaxanthin to chl-*a*, which is primarily responsible for the overall cell colour, was consistent for *S. aurantia* with the reports of small orange cysts in a previous study from Svalbard (Müller et al. 1998). The ratios in *S. nivaloides* were comparable or slightly lower than those previously reported (21:1 from Wyoming, Bidigare et al. 1993; 18–25:1 in Tyrolean Alps, Remias et al. 2005; 34:1 from Svalbard, sample “a8” of Müller et al. 1998). However, red cells from the early melting season had lower astaxanthin-to-chl-*a* ratios (8:1 in Remias et al. 2005; and in Remias and Lütz 2007). The less intense colour of *S. aurantia* can be explained partly by the lower astaxanthin-to-chl-*a* ratio compared to *S. nivaloides*. Theoretically, the total pigment content could be less as well, but a calculation based on dry mass was not possible, because the harvested samples had cryoconite particles attached to the outer cell wall which precluded accurate weights. A further hypothesis why less intense overall pigmentation was observed in *S. aurantia* is because the astaxanthin concentration in their lipid bodies should be lower. On the other hand, the observed differences in the relative contents of *cis*-isomers to all-*trans* astaxanthin between the two species have no impact on cell pigmentation. The absorption peak shift between *cis*- and all-*trans* isomers in the visible range is small (468.0 vs 477.6 nm), so that human eye cannot distinguish it (Yuan and Chen 1997). Consequently, the astaxanthin isomers have the same visual red colour. The main absorption differences take place in the UV-A range. The broad ranges of astaxanthin-to-chl-*a* ratios in samples within both species may be caused by the individual extent of maturation of a population in course of the summer season (e.g. Fig. 3a); however, this should be tested by single-cell metabolomics (Lutz et al. 2015). Also, site-specific factors like exposure to irradiation or the nutrient availability may play a role. However, this was not systematically tested. Since the cysts are metabolically active (as demonstrated by the rapid light curves) but do not divide (cell cleavages of the cysts on the snow surface has never been observed for *Sanguina*, to our knowledge), their fate is to continue accumulating lipids and astaxanthin until the limit of cellular storage capabilities in lipid bodies. The *cis*-isomer of astaxanthin was detected in a smaller amount in Arctic samples using a C30 HPLC column compared to several high alpine populations of red spherical cysts formerly assigned to “*Chlamydomonas nivalis*” from the Austrian Alps measured with a different method using a C18 column (Remias and Lütz 2007).

As far as it is known, there are no further species causing orange snow phenomenon in the (Sub-)Arctic than *S. aurantia*; consequently, this species can be identified by macroscopic observation in these regions, because other snow algae causing a similar snow discoloration, e.g. *C. polyptera* found next to penguin colonies (Remias et al. 2013), are not known from the northern hemisphere.

### Photobiology

The rapid light curves of field cysts differed between the two studied *Sanguina* species: the low light saturation point (*I*_k_) of *S. aurantia* indicated that one third to almost a half of photosynthetic active irradiation (PAR) was sufficient for PS II to become saturated, compared to *S. nivaloides*. Moreover, as apparent from the higher alpha value, *S. aurantia* was more effective in utilising low light levels. Minor photobiological differences between the two *S. nivaloides* populations may be explained by the exposure of their populations to the different cardinal directions (north-western vs. south-eastern exposure of the snowfield) and different maturation level of the cells (i.e. astaxanthin-to-chl-*a* ratio: WP206r vs. WP204 = 11.9 vs. 16.3).

The first insight into the differences in photobiology between orange and red spherical cysts from Svalbard was reported previously using PAM fluorometry in situ (Stibal et al. 2007). Contrary to these Arctic populations, no photoinhibition was noticed up to high light levels of 2000 µmol photons m^−2^ s^−1^, and the alpha was almost twice higher for red spherical cysts from the Austrian Alps (Procházková et al. 2018b) indicating photobiological plasticity of the alpine population at low and high light levels. Since this alpine population was genetically identical to the Arctic one in the ITS2 rDNA molecular marker (haplotype “H1” sensu Procházková et al. 2019a), this finding is indicating an intraspecific flexibility of photosystem II in *S. nivaloides* in adapting to prevailing local light conditions. A similar phenomenon was observed for another snow alga *C. hindakii* (Procházková et al. 2019b). However, there are no data available yet about photobiology and pigment content of *S. aurantia* populations from mid-latitude mountains in Colorado and Washington States. Indeed, high alpine sites significantly differ from the Arctic/Antarctic ones in ambient maximal PAR irradiances, reaching around noon up to ~ 2500 µmol photons m^−2^ s^−1^ in the former (Sommaruga and Psenner 1997; Gorton et al. 2001), while being considerably lower (about 1500 μmol m^−2^ s^−1^) due to lower light angle and frequent cloud cover in the latter (Stibal et al. 2007; Davey et al. 2019). In proximity to our sampling sites at the glacier Longyearbreen, maximal PAR around noon on a summer sunny day was reported to vary between ~ 1000 and 1100 μmol m^−2^ s^−1^ (Remias et al. 2012). The rapid light curves of the polar populations of the two *Sanguina* species indicate shade-adapted photophysiology (higher alpha, relatively low *I*_k_, activity decline from medium light levels on) which may reflect local low light conditions at Svalbard. At King George Island (Antarctica), photosynthetic parameters of snow microbial communities were dependent on sampling site, species composition and bloom colour (Soto et al. 2020). Consistently with our study, their orange snow comprising mainly the OTUs assigned to *Sanguina* spp., uncultured alga OTU 004 and uncultured *Chlorella* spp. had higher alpha and twice lower *I*_k_ when compared to red snow community dominated by *Sanguina* spp. and *C. chenangoensis* (Soto et al. 2020).

## Conclusion

This study provided the first ecophysiological comparison of two closely related species causing snow blooms in the Arctic. Although the cysts of *S. aurantia* and *S. nivaloides* share the same habitats, they significantly differed in cell sizes, astaxanthin-to-chlorophyll-*a* ratios and quantitative fatty acid composition. From a physiological point of view, the restriction of *S. aurantia* to (Sub-)Arctic climates remains unknown but could be attributed to the lower astaxanthin content, leading to less UV absorption capacity compared to the cosmopolitan *S. nivaloides*. Moreover, the formation of plastoglobules reducing the amount of thylakoid membranes in *S. nivaloides* may contribute to photoprotection.

## Supplementary Information

Below is the link to the electronic supplementary material.

Supplementary Information (DOCX 252 kb)
