# Supplementary material for: Ecophysiological and ultrastructural characterisation of the circumpolar orange snow alga Sanguina aurantia compared to the cosmopolitan red snow alga Sanguina nivaloides (Chlorophyta)
Source: Polar Biol. 2020 Dec 11;44(1):105–17. doi: 10.1007/s00300-020-02778-0 (PMC7819945; doi:10.1007/s00300-020-02778-0)
Supplement: Supplementary file 1 — Supplementary Information (DOCX 252 kb) [file 300_2020_2778_MOESM1_ESM.docx]

**Supplemental information**

**Ecophysiological and ultrastructural characterisation of the circumpolar orange snow alga *Sanguina aurantia* compared to the cosmopolitan red snow alga *Sanguina nivaloides* (Chlorophyta)**

*Polar Biology*

Lenka Procházková^1*^, Daniel Remias^2^, Andreas Holzinger^3^,

Tomáš Řezanka^4^ and Linda Nedbalová^1^

^1^Charles University, Faculty of Science, Department of Ecology, Viničná 7, Prague, Czech

Republic,

^2^University of Applied Sciences Upper Austria, School of Engineering, Stelzhamerstr. 23, 4600 Wels, Austria,

^3^Functional Plant Biology, Department of Botany, University of Innsbruck, Sternwartestrasse 15, A-6020 Innsbruck, Austria, https://orcid.org/0000-0002-7745-3978

^4^ Institute of Microbiology, The Czech Academy of Sciences, Vídeňská 1083, 142 20 Prague, Czech Republic, https://orcid.org/0000-0002-8704-9645

^*^ corresponding author: email <lenkacerven@gmail.com>


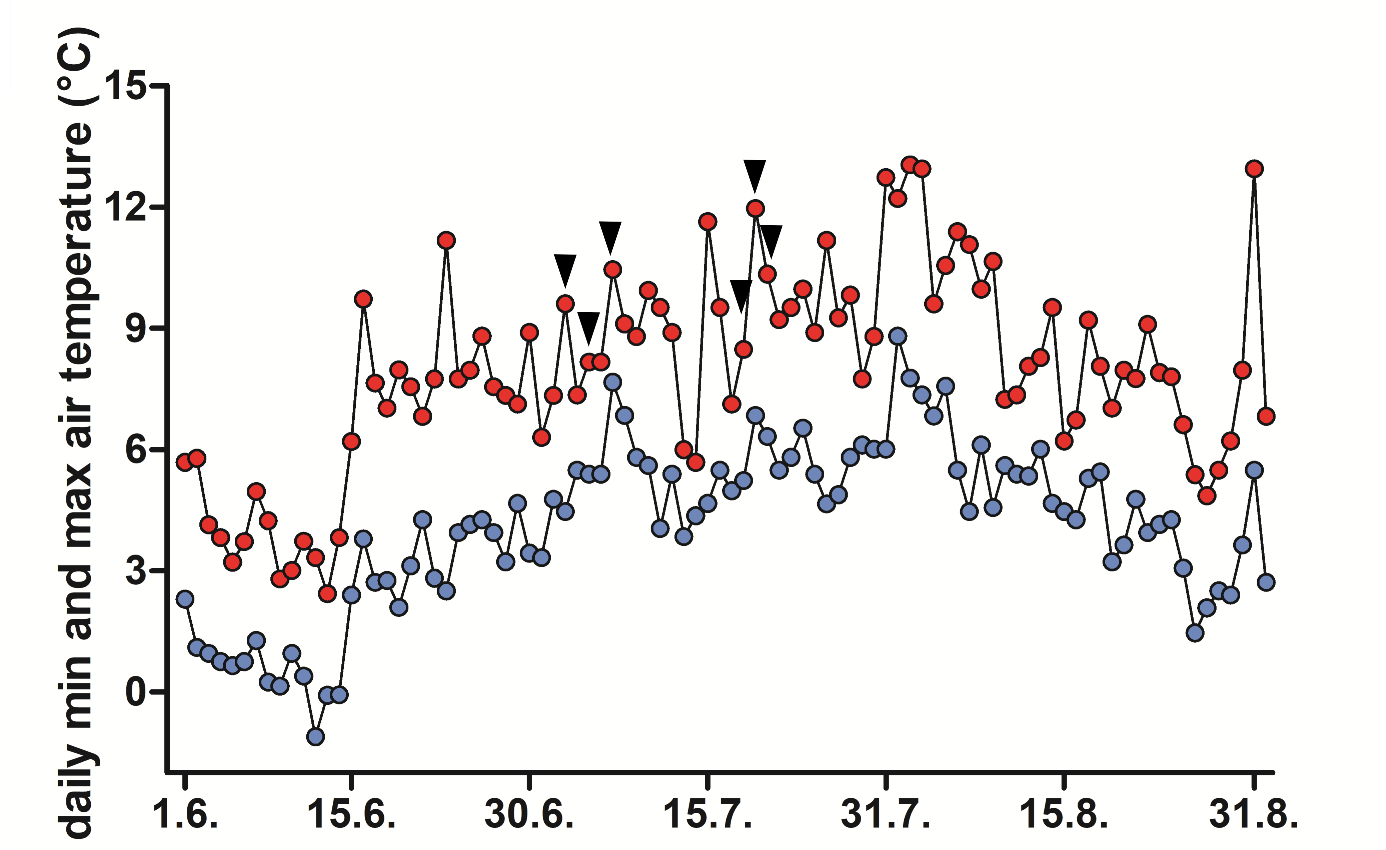


**Online Resource 1** Daily maximum (red) and daily minimum (blue) air temperature in summer period when the *Sanguina aurantia* and *Sangina nivaloides* blooms are most often found in the Arctic (June to August 2018; Adventdalen). Black arrows indicate the dates of the snow harvest in course of this study


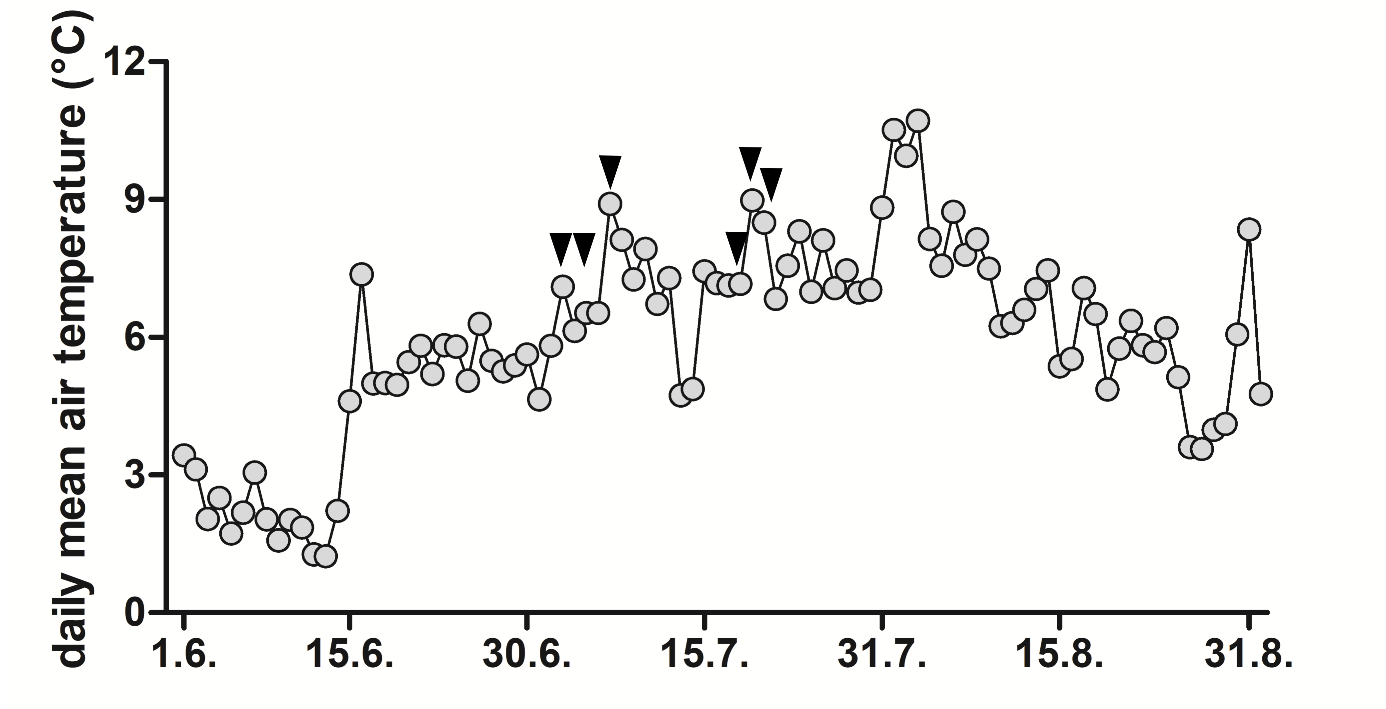


**Online Resource 2** Daily mean air temperature in summer period when the *Sanguina aurantia* and *Sangina nivaloides* blooms are most often found in the Arctic (June to August 2018; Adventdalen). Black arrows indicate the dates of the snow harvest in course of this study

**Online Resource 3** Cellular fatty acid composition of *S. aurantia* and *S. nivaloides* field cysts in [%] of total fatty acids (TL; all samples) and in [%] of the three major lipid classes (samples WP199, WP204): Neutral lipids (NL), phospholipids (PL), and glycolipids (GL). The table shows only fatty acids that had abundances greater than 0.1%. The relative proportion of saturated (SAFA), monounsaturated (MUFA), and polyunsaturated (PUFA) fatty acids is also given.

|  | ***Sanguina aurantia*** | | | | | | | | | | | ***Sanguina nivaloides*** | | | | | | | | | |
| --- | --- | --- | --- | --- | --- | --- | --- | --- | --- | --- | --- | --- | --- | --- | --- | --- | --- | --- | --- | --- | --- |
| **Fatty acids** | **WP199** | | | | **MN13** | | **MN21** | | **WP206o** | | | **WP204** | | | | | | | **MN01** | | **MN22** |
|  | **TL** | **NL** | **PL** | **GL** | | **TL** | | **TL** | | **TL** | **TL** | | | **NL** | | **PL** | | **GL** | | **TL** | **TL** |
| 14:0 | 0.9 | 1.0 | 1.3 | 0.4 | | 0.6 | | 7.8 | | 0.7 | 0.1 | | | 0.1 | | 0 | | 0 | | 0.2 | 0.9 |
| 15:0 | 0.1 | 0.1 | 0 | 0 | | 0.2 | | 0.1 | | 0.2 | 0 | | | 0 | | 0 | | 0 | | 0 | 0.1 |
| 16:0 | 23.6 | 16.0 | 23.0 | 38.6 | | 11.5 | | 10.8 | | 16.3 | 8.9 | | | 6 | | 25.1 | | 9.8 | | 6.8 | 7.9 |
| 16:1(11Z) | 0 | 0 | 0 | 0 | | 0 | | 0.1 | | 0 | 0 | | | 0 | | 0 | | 0 | | 0 | 0 |
| 16:1 (9Z) | 1.2 | 0.2 | 6.8 | 0.0 | | 0.4 | | 1.4 | | 0.7 | 0 | | | 0 | | 0.1 | | 0 | | 0 | 0.2 |
| 16:1 (7Z) | 0.5 | 0.7 | 0.7 | 0.1 | | 2.3 | | 1.8 | | 3.6 | 1.7 | | | 2 | | 0.6 | | 1.4 | | 1.2 | 2.0 |
| 16:3 (7Z,10Z,13Z) | 1.3 | 1.5 | 2.5 | 0.2 | | 1.5 | | 0.5 | | 0.7 | 3 | | | 3.4 | | 1.9 | | 1.8 | | 3.5 | 3.8 |
| 16:4 (4Z,7Z,10Z,13Z) | 6.9 | 10.3 | 5.0 | 1.4 | | 4.3 | | 2.1 | | 1.9 | 11.1 | | | 11.3 | | 9.7 | | 11.4 | | 8.3 | 7.0 |
| 17:0 | 0.1 | 0.1 | 0.1 | 0.1 | | 0.3 | | 0.2 | | 0.4 | 0.1 | | | 0.1 | | 0.1 | | 0.1 | | 0.1 | 0.2 |
| 18:0 | 23.3 | 8.2 | 20.5 | 54.2 | | 38.5 | | 30.4 | | 49.2 | 7.3 | | | 6.8 | | 3 | | 13 | | 18.2 | 11.8 |
| 18:1 (11Z) | 0.8 | 0.6 | 2.7 | 0.1 | | 1.8 | | 0.0 | | 1.2 | 2.1 | | | 1.6 | | 5.8 | | 1.4 | | 1.2 | 2.6 |
| 18:1 (9Z) | 22.5 | 35.8 | 13.3 | 2.3 | | 17.3 | | 27.1 | | 11.9 | 26.3 | | | 29.8 | | 8.1 | | 24.4 | | 25.2 | 27.6 |
| 18:2 (9Z,12Z) | 6.2 | 8.9 | 7.1 | 0.5 | | 6.6 | | 4.3 | | 6.1 | 18.7 | | | 21.1 | | 12.5 | | 12.3 | | 13.3 | 12.1 |
| 18:3 (9Z,12Z,15Z) | 9.5 | 12.2 | 14.7 | 1.3 | | 8.4 | | 2.6 | | 3.6 | 13.8 | | | 12.1 | | 21.7 | | 15.7 | | 14.8 | 16.0 |
| 18:4 (6Z,9Z,12Z,15Z) | 1.5 | 2.0 | 1.8 | 0.3 | | 2.3 | | 6.0 | | 1.1 | 6.3 | | | 5.2 | | 10.7 | | 8.2 | | 4.0 | 6.0 |
| 19:0 | 0 | 0 | 0 | 0 | | 0 | | 0 | | 0.1 | 0 | | | 0 | | 0 | | 0 | | 0 | 0 |
| 19:3 (7Z,10Z,13Z) | 0 | 0 | 0 | 0 | | 0.1 | | 0.6 | | 0.1 | 0.1 | | | 0 | | 0.6 | | 0 | | 0 | 0.1 |
| 20:0 | 0.8 | 1.1 | 0.2 | 0.4 | | 1.4 | | 0.7 | | 1.1 | 0.5 | | | 0.5 | | 0.1 | | 0.5 | | 2.2 | 0.9 |
| 20:2 (11Z, 14Z) | 0 | 0 | 0 | 0 | | 0 | | 0 | | 0 | 0 | | | 0 | | 0 | | 0 | | 0.1 | 0 |
| 20:3 (11Z,14Z,17Z) | 0 | 0 | 0 | 0 | | 0.1 | | 0.6 | | 0 | 0 | | | 0 | | 0 | | 0 | | 0.2 | 0.2 |
| 20:3 (8Z,11Z,14Z) | 0 | 0 | 0 | 0 | | 0.0 | | 0.2 | | 0 | 0 | | | 0 | | 0 | | 0 | | 0 | 0 |
| 20:4 (8Z,11Z,14Z,17Z) | 0 | 0 | 0 | 0 | | 0.6 | | 0.9 | | 0.1 | 0 | | | 0 | | 0 | | 0 | | 0.1 | 0.2 |
| 20:4 (5Z,8Z,11Z,14Z) | 0 | 0 | 0 | 0 | | 0.1 | | 0.2 | | 0 | 0 | | | 0 | | 0 | | 0 | | 0 | 0 |
| 20:5 (5Z,8Z,11Z,14Z,17Z) | 0 | 0 | 0 | 0 | | 0.7 | | 1.1 | | 0.2 | 0 | | | 0 | | 0 | | 0 | | 0 | 0.1 |
| 22:0 | 0.7 | 1.2 | 0.1 | 0.1 | | 0.9 | | 0.4 | | 0.7 | 0 | | | 0 | | 0 | | 0 | | 0.6 | 0.2 |
| 23:0 | 0.1 | 0.1 | 0 | 0 | | 0 | | 0 | | 0 | 0 | | | 0 | | 0 | | 0 | | 0 | 0 |
| SAFA | 49.6 | 27.8 | 45.3 | 93.8 | | 53.4 | | 50.5 | | 68.6 | 16.9 | | 13.5 | | 28.3 | | 23.4 | | 28.1 | | 22.0 |
| MUFA | 25.0 | 37.3 | 23.5 | 2.5 | | 21.9 | | 30.4 | | 17.4 | 30.1 | | 33.4 | | 14.6 | | 27.2 | | 27.6 | | 32.4 |
| PUFA | 25.4 | 34.9 | 31.2 | 3.7 | | 24.7 | | 19.2 | | 13.8 | 53.0 | | 53.1 | | 57.1 | | 49.4 | | 44.3 | | 45.5 |
